# Establishment and validation of PTE prediction model in patients with cerebral contusion

**DOI:** 10.1038/s41598-022-24824-z

**Published:** 2022-11-29

**Authors:** Shengwu Lin, Qianqian Wang, Yufeng Zhu, Xiaoqing Jin, Pei Han, Zhongsheng Lu

**Affiliations:** 1 Department of Graduate School, Qinghai University, Xining, 810016 Qinghai China; 2 Department of Neurosurgery, Qinghai Provincial People’s Hospital, Xining, 810007 Qinghai China

**Keywords:** Medical research, Neurology, Risk factors

## Abstract

Post-traumatic epilepsy (PTE) is an important cause of poor prognosis in patients with cerebral contusions. The primary purpose of this study is to evaluate the high-risk factors of PTE by summarizing and analyzing the baseline data, laboratory examination, and imaging features of patients with a cerebral contusion, and then developing a Nomogram prediction model and validating it. This study included 457 patients diagnosed with cerebral contusion who met the inclusion criteria from November 2016 to November 2019 at the Qinghai Provincial People's Hospital. All patients were assessed for seizure activity seven days after injury. Univariate analysis was used to determine the risk factors for PTE. Significant risk factors in univariate analysis were selected for binary logistic regression analysis. *P* < 0.05 was statistically significant. Based on the binary logistic regression analysis results, the prediction scoring system of PTE is established by Nomogram, and the line chart model is drawn. Finally, external validation was performed on 457 participants to assess its performance. Univariate and binary logistic regression analyses were performed using SPSS software, and the independent predictors significantly associated with PTE were screened as Contusion site, Chronic alcohol use, Contusion volume, Skull fracture, Subdural hematoma (SDH), Glasgow coma scale (GCS) score, and Non late post-traumatic seizure (Non-LPTS). Based on this, a Nomogram model was developed. The prediction accuracy of our scoring system was C-index = 98.29%. The confidence interval of the C-index was 97.28% ~ 99.30%. Internal validation showed that the calibration plot of this model was close to the ideal line. This study developed and verified a highly accurate Nomogram model, which can be used to individualize PTE prediction in patients with a cerebral contusion. It can identify individuals at high risk of PTE and help us pay attention to prevention in advance. The model has a low cost and is easy to be popularized in the clinic. This model still has some limitations and deficiencies, which need to be verified and improved by future large-sample and multicenter prospective studies.

## Introduction

Cerebral contusion is one of the most severe types of traumatic brain injury (TBI), occurring in 20–30% of patients with TBI^1^, resulting in death, disability, and reduced quality of life. Post-traumatic epilepsy (PTE) is one of the most disabling complications among surviving patients with cerebral contusion and may be challenging to treat^2^. With further population growth in China, the absolute number of patients with cerebral contusions will also exceed that of most other countries. At the same time, with the improvement in medical level, more and more patients are expected to survive severe cerebral contusion, which increases the number of complications related to cerebral contusion.

Post-traumatic seizure (PTS) is a potential sequela of cerebral contusion, accounting for 5% of all seizures and 20% structural epilepsy^3,4^. A previous study^5^ defined PTS as a single non-recurrent convulsive and divided it into three types according to the time of occurrence: 1. Immediate post-traumatic seizure (IPTS) refers to seizures that occur within 24 h after injury; 2. Early post-traumatic seizure (EPTS) refers to seizures that occur more than 24 h and within seven days of injury; 3. Late post-traumatic seizure (LPTS) is defined as epileptic seizures more than one week after trauma. With the new definition of epilepsy proposed by the International League Against Epilepsy (ILAE) and the International Bureau of Epilepsy (IBE), PTE is defined as one or more recurrent late seizures occurring (including LPTS) more than one week after trauma^6^. Some studies have suggested that IPTS and EPTS are directly related to the primary injury, and late seizures are attributed to secondary cascade injury and persistent Epilepsy induced mechanism^7^.

In patients with a cerebral contusion, the incubation period for the first episode of PTE is usually several weeks to several years. All previous trials of antiepileptic drug therapy to prevent PTE in humans have been unsuccessful due to the difficulty of identifying people at high risk of epileptic seizures after TBI^8^. It is generally believed that treatment of early post-traumatic epilepsy does not affect the incidence of PTE and that routine prophylactic antiepileptic drugs do not reduce the mortality or disability caused by PTE^7^. Although prophylactic antiepileptic therapy does not reduce PTE incidence, it may reduce acute episodes and, by reducing secondary brain damage, is expected to prolong PTE latency and thus improve PTE prognosis^9^. Therefore, we hope to objectively predict the individual risk of epileptic seizures by finding out the risk factors associated with PTE in combination with the clinical characteristics of patients.

In many previous studies, the main risk factors for PTE were considered to include: age, the severity of cerebral contusion, post-traumatic amnesia, epidural hematoma (EDH), subdural hematoma (SDH), loss of consciousness (LOC) > 30 min, skull fracture, IPTS and EPTS and so on. However, different studies provide different results^3,10–16^. Therefore, in this study, we collected baseline information and clinical data from patients with cerebral contusion admitted to Qinghai Provincial People's Hospital over the past three years. Screened independent risk factors leading to PTE development in patients with a cerebral contusion, established a Nomogram model and validated it. It is hoped that this Nomogram will be used to identify the high-risk group of cerebral contusion patients who will develop PTE. According to the research on these high-risk PTE patients, the antiepileptic treatment of PTE will be possible in the future.

## Methods

### Study patients

**Figure 1:**
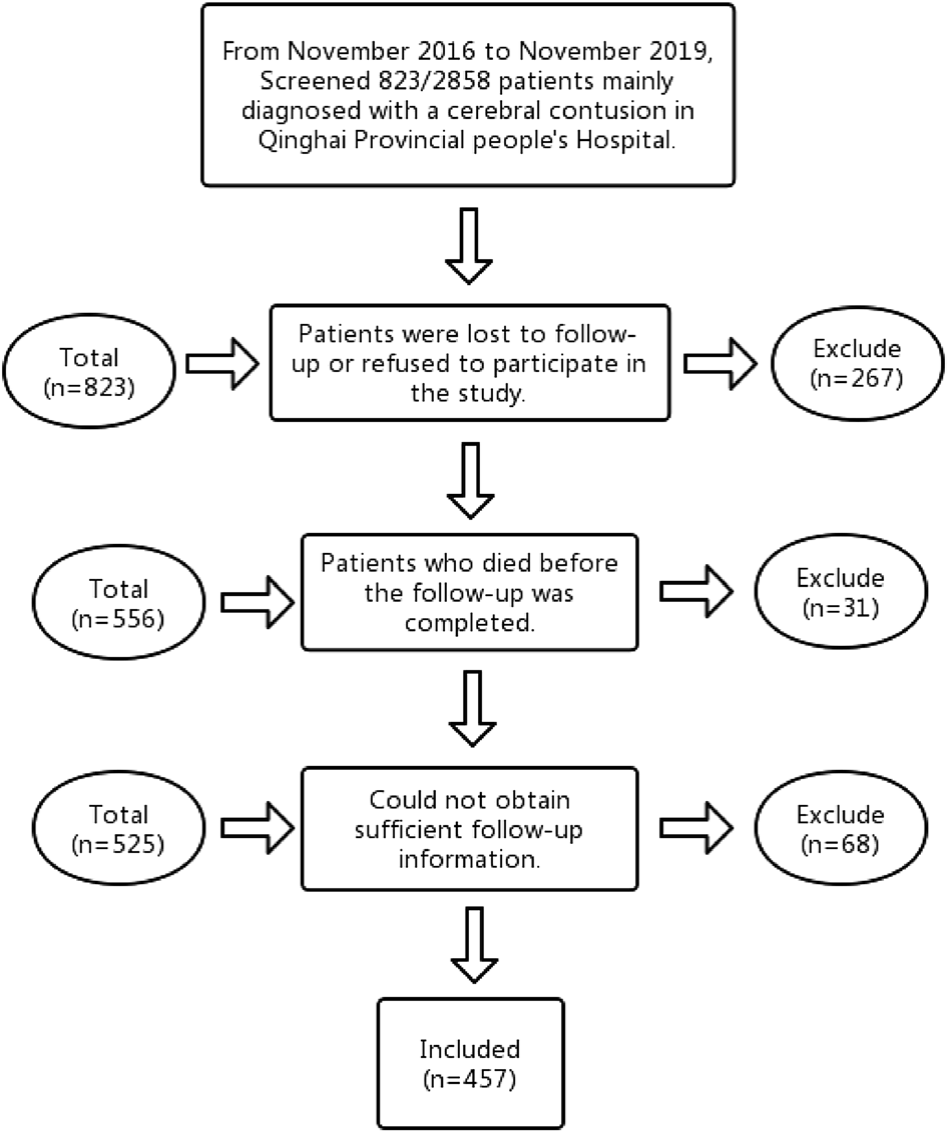
Patient screening flow chart.

In order to ensure that the clinical observation of PTE is more than two years, we retrospectively analyzed the baseline and clinical data of all patients with cerebral contusion admitted to Qinghai Provincial People's Hospital from November 2016 to November 2019. In this study, 823 patients with cerebral contusion were selected from 2858 patients with brain trauma. According to the inclusion and exclusion criteria, we excluded 336 of them. The reasons are as follows: 267 patients lost follow-up, 31 patients died before the completion of follow-up, and 68 patients could not obtain sufficient follow-up information. In the end, 457 participants were left for final analysis (Fig. 1).

### Inclusion and exclusion criteria

Inclusion criteria: (1) those aged ≥ 18 years; (2) the cranial injury was due to external forces and the primary clinical diagnosis was cerebral contusion; (3) the traumatic event occurred from November 2016 to November 2019; (4) there was complete trauma-related information in the medical records; (5) the patients had been treated at Qinghai Provincial People's Hospital, and all patients with cerebral contusion were treated in strict accordance with neurosurgical treatment guidelines.

Exclusion criteria: (1) patients known to have epilepsy prior to cerebral contusion; (2) patients with cerebrovascular disease, encephalitis, brain tumors, drug abuse, and other chronic diseases that can cause seizures; (3) During the follow-up, other conditions might lead to seizures before the appearance of PTE; (4) Patients or their family members refuse to participate in the study; (5) Patients with a combination of other serious underlying diseases; (6) The required clinical data and imaging data are incomplete.

### Ethics approval

Due to the clinical observation time of patients in our study being more than two years, the long time since patients were discharged from the hospital, and the limitations of geographical location or other factors after discharge, all patients (or their agents) could not sign written informed consent before participating in the study. Therefore, we can only obtain their oral informed consent by telephone inquiry. The Ethics Committee has approved this study of Qinghai People's Hospital (the reference number for the ethics approval is 2022–42), and the ethics committee deemed that oral informed consent was sufficient for this study. The study was conducted under the ethical standards in the Helsinki Declaration (2013 revision of Brazil). All methods were carried out according to the relevant guidelines and regulations.

## Data extraction

### Clinical data

The clinical data of the patients included: sex, age, ethnicity, injured type, Chronic alcohol use, High blood pressure (HBP), Diabetes mellitus (DM), Admission Glasgow coma scale (GCS) score, Intracranial pressure (ICP), Blood glucose, Cerebrospinal fluid glucose/Cerebrospinal fluid lactate ratio (C-G/L), surgery, Traumatic coagulation abnormalities, Intracranial infections (ICI), hospital stay, Non-LPTS (IPTS + EPTS), Glasgow outcome scale (GOS) score at discharge, and so on (Table 2). The blood glucose and blood coagulation indexes were extracted from the biochemical and coagulation tests within 24 h after admission.

### Imaging data

All CT examinations were performed using a unified standard (axial thickness 5 mm). The data we provided from craniocerebral CT included: Contusion site, Contusion volume, Skull fracture, EDH, SDH, Subarachnoid hemorrhage (SAH), Traumatic brainstem hemorrhage (TBH), Diffuse axonal injury (DAI), Midline shift (MLS), Cerebral hernia, Third ventricle status (TVS), and so on (Table 2). These CT images and data are available from our hospital computers' CT image viewing software. All data for each patient is extracted by two experienced clinicians and re-extracted if there are differences until the agreement is finally reached.

#### Measurement of contusion volume

**Figure 2:**
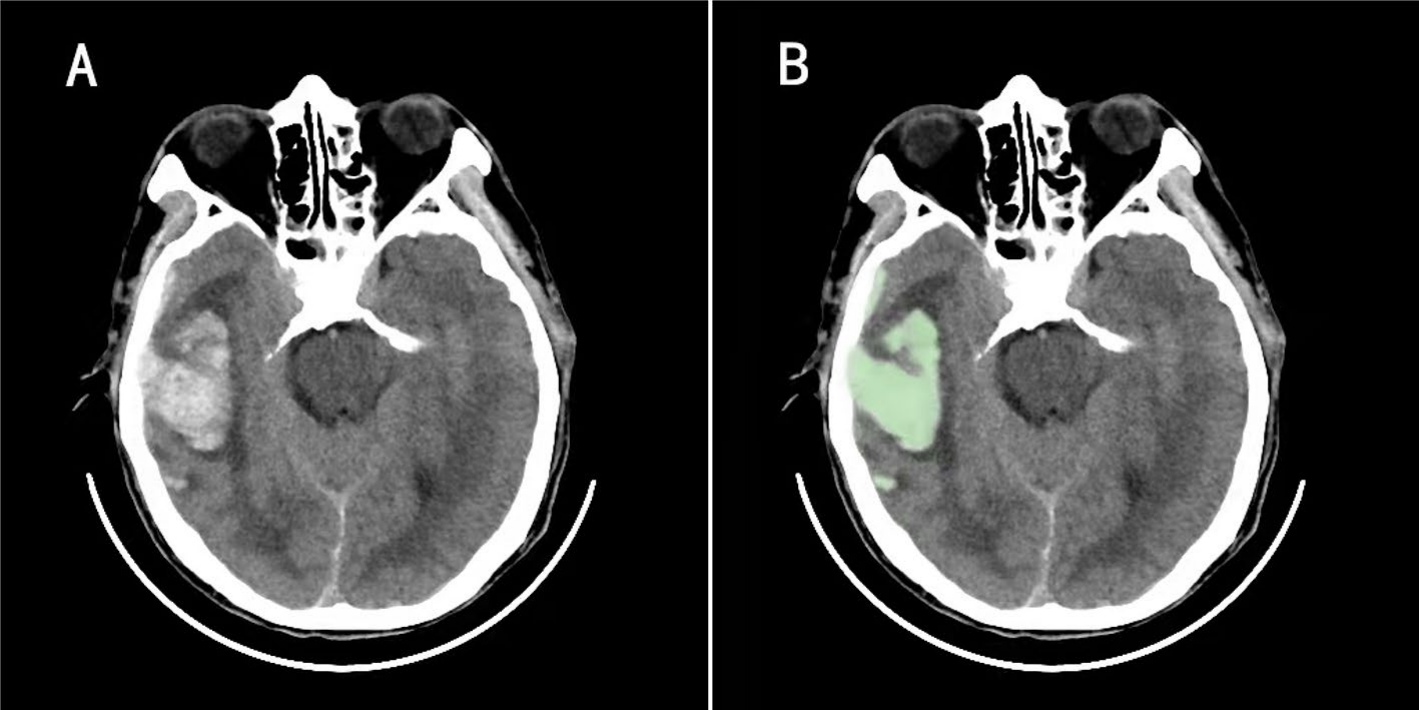
On the 3D slicer software, the contusion volume is provided by manually selecting the region of interest, setting the threshold based on the Hounsfield unit (the fixed thresholds of 110 and 50 HU) to distinguish the contusion part from the surrounding normal brain tissue, and automatically summarizing the adjacent voxels (A and B).

Extracting the patient's cranial CT data within 6 h of admission and measuring the contusion volume by computer-aided software 3D Slicer (version 4.8.0; Harvard University, New York). 3D slicer provides contusion volumes by manually selecting the region of interest, setting thresholds based on Hounsfield units (fixed windows of 110 and 50 HU) to distinguish between the contused portion of the brain and the surrounding normal brain tissue, and automatically summarizing adjacent voxels (Fig. 2A and B). When multiple contusions were present, the total volume was calculated.

### Collection of follow-up data

Our findings measure the presence of epileptic waveforms in patients on electroencephalography (EEG) tests 7 days after the onset of brain contusion. All patients with brain contusions were followed up by a properly trained neurosurgeon by telephone after discharge. During the telephone follow-up, if the patient and his or her family agree to participate in the survey, data on the occurrence of PTE during the patient's hospitalization can be obtained directly from the patient's "medical documentation record" during the hospitalization. If the patient's PTE occurs after discharge, or if the patient has symptoms similar to epilepsy occurrence but has not been clearly diagnosed, we will follow up with a validated questionnaire and then record in detail the patient's general condition, the circumstances and frequency of epileptic symptoms at the time of occurrence, the EEG results during the re-visit, and the treatment of the seizure. Moreover, after the patient or his family reported the occurrence of PTE or symptoms similar to epilepsy, our team of neurosurgeons will speak with them in person and further determine the diagnosis of PTE based on the patient's clinical presentation and EEG results.

## Statistical analysis

SPSS software (USA.IBM.24.0) was used for univariate and binary logic regression analysis. In this study, P-value, odds ratio, and 95% confidence interval were used to evaluate all factors related to PTE. Chi-square and Fisher exact tests were used to determine risk factors for univariate analysis. Then the binary logic regression analysis was carried out according to the significant risk factors in the univariate analysis. When *P* < 0.05, we think it has statistical significance. Based on the binary logic regression analysis results, the forest map was drawn by Graphpad software (Version 9.0.0 for Windows, GraphPad Software, San Diego, California USA), and the line chart model was drawn according to the comprehensive score.

## Results

### Division of critical values

In order to establish the scoring system of PTE, continuous variables need to be graded first. However, there is no theoretical basis for grading continuous variables such as age, length of stay, etc. Therefore, in this study, the ROC curve is selected to divide the critical value of continuous variables (Table 1).

**Table 1:** Division of critical value.

	Auc	*P*	Cutoff
Age	0.516	0.700	> 52
Volume	0.896	< 0.001	> 13.5
MLS	0.555	0.1433	> 1.8
ICP	0.725	< 0.001	> 250
Blood glucose	0.661	< 0.001	> 6.78
C-G/L	0.656	< 0.001	≤ 5.17
ICU length of stay	0.786	< 0.001	> 0
Hospital length of stay	0.741	< 0.001	> 26

There is no theoretical basis for the grading of continuous variables such as Age, Volume, MLS, ICP, Blood glucose, C-G/L, and Length of stay. Thus, the ROC curve is selected to divide the critical value of continuous variables.

*MLS* midline shift, *ICP* intracranial pressure, *C-G/L* cerebrospinal fluid glucose/cerebrospinal fluid lactate ratio.

### Analysis of influencing factors of PTE

In order to predict PTE in patients, factors such as age, gender, and site of cerebral contusion were initially considered suspicious factors for PTE based on practical experience. In this study, we screened the influential factors that can cause PTE by statistical methods and used these factors to establish a scoring system for PTE, which is of great practical significance. Due to the establishment of the PTE scoring system, the requirement for accuracy is relatively high. Therefore, to ensure the accuracy and rigor of the study, the significance level chosen is *P* = 0.05.

#### Univariate analysis

First of all, for suspicious factors. In order to avoid the mixed interference among factors, it is necessary to conduct a preliminary screening of these factors, that is, a single-factor analysis. The essence of univariate analysis is to compare whether there are significant differences in PTE among various types under a certain factor. And if the difference in PTE rate is statistically significant, then the factor is retained, and the subsequent multi-factor analysis is continued. If the multi-factor is significant, participate in establishing the PTE scoring system.

The results of the univariate analysis of the suspicious factors are shown in Table 2: It can be seen that at a significance level of 0.05, Age, Sex, Contusion site, Chronic alcohol use, Contusion volume, Fracture, SDH, SAH, TVS, GCS score, ICP, Blood glucose, C-G/L, ICI, ICU length of stay, Hospital length of stay, Non-LPTS, and GOS score were significantly different in the incidence of PTE. Therefore these factors need to be screened out for subsequent multifactorial analysis and establishing an index system.

**Table 2:** Univariate analysis results (statistically significant).

	PTE		Incidence of PTE (%)	*χ*^2^	*P*
	No	Yes			
**Age**					
≤ 52	289	45	13.5	4.025	0.045
> 52	97	26	21.1		
**Sex**					
Male	310	64	17.1	3.899	0.048
Female	76	7	8.4		
**Ethnicity**					
Han	225	49	17.9	7.49	0.058
Tibetan	88	8	8.3		
Hui	41	11	21.2		
other	32	3	8.6		
**Injured type**					
Traffic	129	25	16.2	2.365	0.5
Violence	36	5	12.2		
Fall	107	15	12.3		
Other	114	26	18.6		
**Site**					
Frontal	111	24	17.8	47.834	< 0.001
Temporal	99	12	10.8		
Parietal	58	1	1.7		
Occipital	58	1	1.7		
Multiple sites	60	33	35.5		
**Chronic alcohol use**					
No	292	33	10.2	24.839	< 0.001
Yes	94	38	28.8		
**HBP**					
No	332	62	15.7	0.087	0.768
Yes	54	9	14.3		
**DM**					
No	367	70	16	1.769	0.183
Yes	19	1	5		
**Volume**					
≤ 13.5	311	13	4	112.664	< 0.001
> 13.5	75	58	43.6		
**Fracture**					
No	245	35	12.5	9.812	0.007
Liner	124	27	17.9		
Depressed	17	9	34.6		
**EDH**					
No	291	53	15.4	0.018	0.894
Yes	95	18	15.9		
**SDH**					
No	323	18	5.3	107.717	< 0.001
Yes	63	53	45.7		
**SAH**					
No	131	13	9	6.787	0.009
Yes	255	58	18.5		
**TBH**					
No	385	70	15.4	–	0.287*
Yes	1	1	50		
**DAI**					
No	381	70	15.5	–	1*
Yes	5	1	16.7		
**MLS**					
≤ 1.8	179	25	12.3	3.023	0.082
> 1.8	207	46	18.2		
**TVS**					
No	344	27	7.3	102.466	< 0.001
Yes	42	44	51.2		
**Therapy**					
Conservative	300	46	13.3	5.494	0.064
Decompressive craniectomy	80	23	22.3		
Postoperative bone flap reduction	6	2	25		
**GCS score**					
13–15 (mild)	353	24	6.4	161.723	< 0.001
9–12 (moderate)	26	18	40.9		
3–8 (severe)	7	29	80.6		
**ICP**					
≤ 250	300	27	8.3	46.417	< 0.001
> 250	86	44	33.8		
**Traumatic coagulation abnormalities**					
No	383	71	15.6	–	1*
Yes	3	0	0		
**Blood glucose**					
≤ 6.78	236	20	7.8	26.46	< 0.001
> 6.78	150	51	25.4		
**C-G/L**					
> 5.17	277	28	9.2	28.229	< 0.001
≤ 5.17	109	43	28.3		
**Cerebral hernia**					
No	381	68	15.1	–	0.113*
Yes	5	3	37.5		
**ICI**					
No	383	65	14.5	–	0.001*
Yes	3	6	66.7		
**ICU length of stay**					
0	373	28	7	182.454	< 0.001
> 0	13	43	76.8		
**Hospital length of stay**					
≤ 26	327	30	8.4	63.253	< 0.001
> 26	59	41	41		
**Non-LPTS**					
No	373	31	7.7	164.122	< 0.001
Yes	13	40	75.5		
**GOS score**					
Vegetative state	3	3	50	61.36	< 0.001*
Severe disability	9	10	52.6		
Mild disability	14	19	57.6		
Good recovery	360	39	9.8		

There were significant differences in the incidence of PTE between different Age, Sex, Contusion site, Chronic alcohol use, Contusion volume, Fracture, SDH, SAH, TVS, GCS score, ICP, Blood glucose, C-G/L, ICI, ICU length of stay, Hospital length of stay, Non-LPTS, and GOS score (*P* < 0.05).

*HBP* high blood pressur, *DM* diabetes mellitus, *EDH* epidural hematoma, *SDH* subdural hematoma, *SAH* subarachnoid hemorrhage, *TBH* Traumatic Brainstem Hemorrhage, *DAI* diffuse axonal injury, *MLS* midline shift, *TVS* third ventricle status, *GCS* glasgow coma scale, *ICP* intracranial pressure, *C-G/L* cerebrospinal fluid glucose/cerebrospinal fluid lactate ratio, *ICI* intracranial infections, *LPTS* late post-traumatic seizure, *COS* glasgow outcome scale.

*Fisher’s exact test.

#### Multivariate analysis

Using the above-screened indicators with significant differences in PTE incidence, we continued to select binary logistic regression for multifactorial analysis. The results are shown in Table 3: Contusion site could significantly affect the incidence of PTE (*P* < 0.05). The OR of parietal and occipital was 0.018 and 0.011, respectively, which indicated that PTE incidence in parietal and occipital was significantly lower than that in frontal. Chronic alcohol use could significantly affect PTE (*P* < 0.05). The OR was 7.442, indicating that PTE incidence in patients with chronic alcohol use is significantly higher than in patients without Chronic alcohol use. Contusion Volume could significantly affect PTE (*P* < 0.05). The OR was 6.566, indicating that PTE incidence in patients with a Contusion Volume > 13.5 ml was significantly higher than in patients with a Contusion Volume ≤ 13.5 ml. Fracture significantly affected PTE (*P* < 0.05). The OR was 25.562, indicating that PTE incidence in patients with skull depression was significantly higher than in patients with No Skull fracture. SDH could significantly affect PTE (*P* < 0.05). The OR was 14.305, indicating that PTE's incidence with SDH was significantly higher than that of patients without SDH. GCS score could significantly affect PTE (*P* < 0.05). The OR was 34.193, indicating that PTE incidence in patients with severe cerebral contusion was significantly higher than in patients with a mild cerebral contusion. Non-LPTS significantly affected PTE (*P* < 0.05). The OR was 78.877, indicating that PTE's incidence with Non-LPTS was significantly higher than patients without Non-LPTS. The other indexes did not significantly affect the incidence of PTE (*P* > 0.05).

**Table 3:** Multivariate analysis.

	B	SE	Wald	P	OR	95% C.I. for OR	
						Lower	Upper
**Age**							
> 52	− 0.233	0.854	0.074	0.785	0.792	0.148	4.225
≤ 52							
**Sex**							
Female	0.559	1.087	0.265	0.607	1.75	0.208	14.722
Male							
			15.051	**0.005**			
**Site**							
Temporal	0.697	0.842	0.684	0.408	2.008	0.385	10.464
Parietal	− 4.001	1.587	6.356	0.012	0.018	0.001	0.41
Occipital	− 4.482	1.962	5.216	0.022	0.011	< 0.001	0.53
Multiple sites	1.168	0.847	1.902	0.168	3.216	0.611	16.918
Frontal							
**Chronic alcohol use**							
Yes	2.007	0.799	6.311	**0.012**	7.442	1.554	35.626
No							
**Volume**							
> 13.5	1.882	0.847	4.941	**0.026**	6.566	1.249	34.511
≤ 13.5							
			6.86	**0.032**			
**Fracture**							
Liner	0.431	0.697	0.382	0.537	1.538	0.392	6.035
Depressed	3.241	1.268	6.531	0.011	25.562	2.129	306.975
No							
**SDH**							
Yes	2.661	0.824	10.436	**0.001**	14.305	2.847	71.872
No							
**SAH**							
Yes	− 0.468	0.829	0.318	0.573	0.627	0.123	3.181
No							
**TVS**							
Yes	1.694	1.013	2.794	0.095	5.44	0.747	39.643
No							
			7.193	**0.027**			
**GCS score**							
9–12 (moderate)	0.855	0.853	1.006	0.316	2.351	0.442	12.502
3–8 (severe)	3.532	1.317	7.192	0.007	34.193	2.588	451.81
13–15 (mild)							
**ICP**							
> 250	− 0.289	0.676	0.183	0.669	0.749	0.199	2.819
≤ 250							
**Blood glucose**							
> 6.78	0.325	0.722	0.203	0.652	1.384	0.336	5.696
≤ 6.78							
**C-G/L**							
> 5.17	0.529	0.761	0.483	0.487	1.697	0.382	7.536
≤ 5.17							
**ICI**							
Yes	2.152	1.349	2.542	0.111	8.599	0.611	121.084
No							
**ICU length of stay**							
> 0	1.432	0.843	2.886	0.089	4.186	0.803	21.832
0							
**Hospital length of stay**							
> 26	− 0.163	0.984	0.027	0.868	0.85	0.124	5.84
≤ 26							
**Non-LPTS**							
Yes	4.368	0.892	23.968	** < 0.001**	78.877	13.725	453.284
No							
GOS score	− 0.907	0.59	2.36	0.124	0.404	0.127	1.284
Constant	− 3.666	2.858	1.645	0.2	0.026		

As can be seen from the above table, Site, Chronic alcohol use, Volume, Fracture, SDH, GCS score, and Non-LPTS can significantly affect PTE (*P* < 0.05).

*SDH* subdural hematoma, *SAH* subarachnoid hemorrhage, *TVS* third ventricle status, *GCS* glasgow coma scale, *ICP* intracranial pressure, *C-G/L* cerebrospinal fluid glucose/cerebrospinal fluid lactate ratio, *ICI* intracranial infections, *LPTS* late post-traumatic seizure, *COS* glasgow outcome scale.

Significant values are in bold.

**Figure 3:**
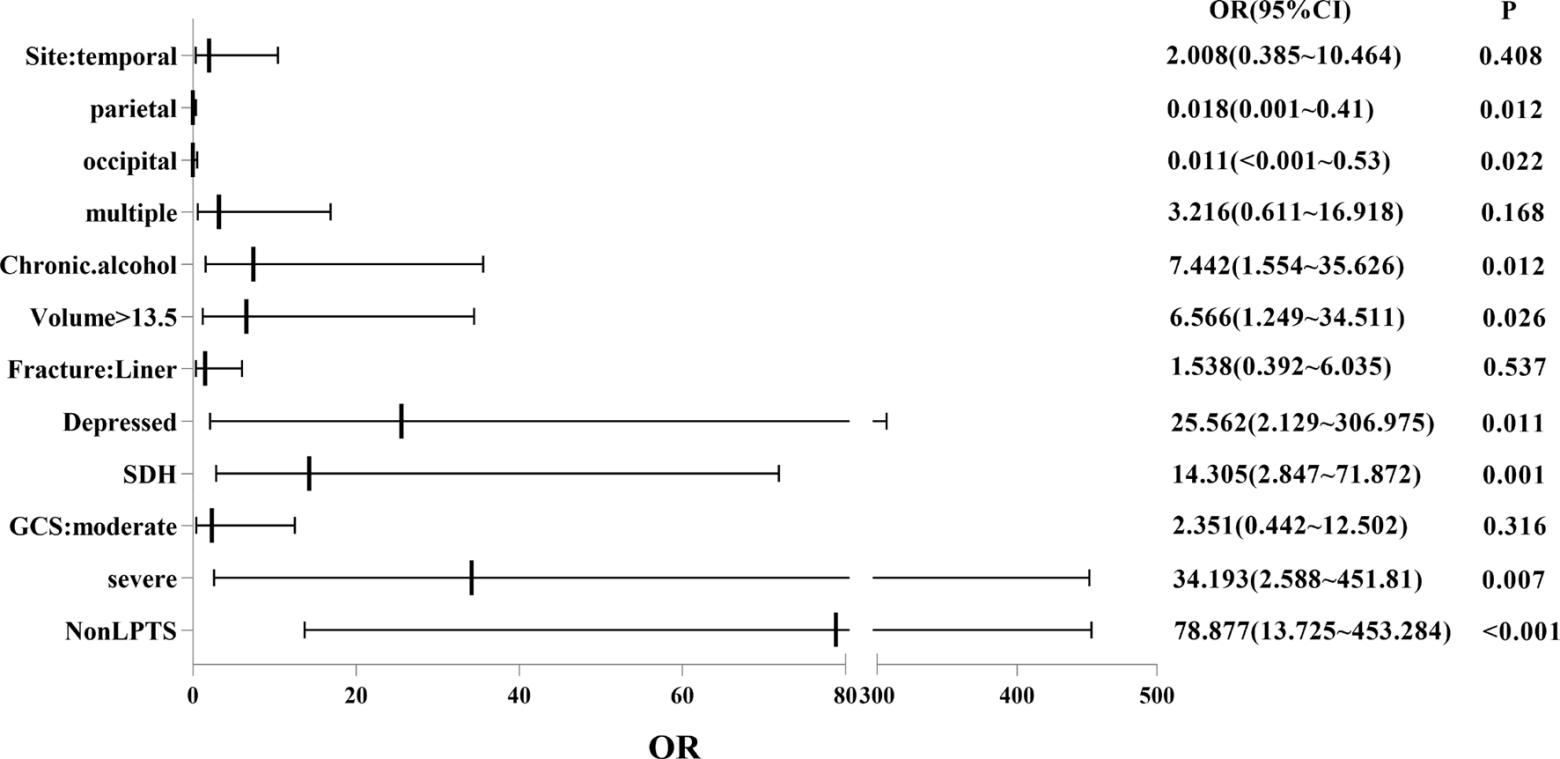
Multivariate analysis was performed for Site, Chronic alcohol use, Volume, Fracture, SDH, GCS score, and Non-LPTS. *CI* confidence interval, *SDH* subdural hematoma, *GCS* glasgow coma scale, *LPTS* late post-traumatic seizure.

In order to see more clearly the dangerous effects of Site, Chronic alcohol use, Volume, Fracture, SDH, GCS, and Non-LPTS on PTE, the OR values of each risk factor are shown in the following forest map (Fig. 3).

### Establishment of nomogram and prediction of PTE incidence

#### Establishment of nomogram

**Figure 4:**
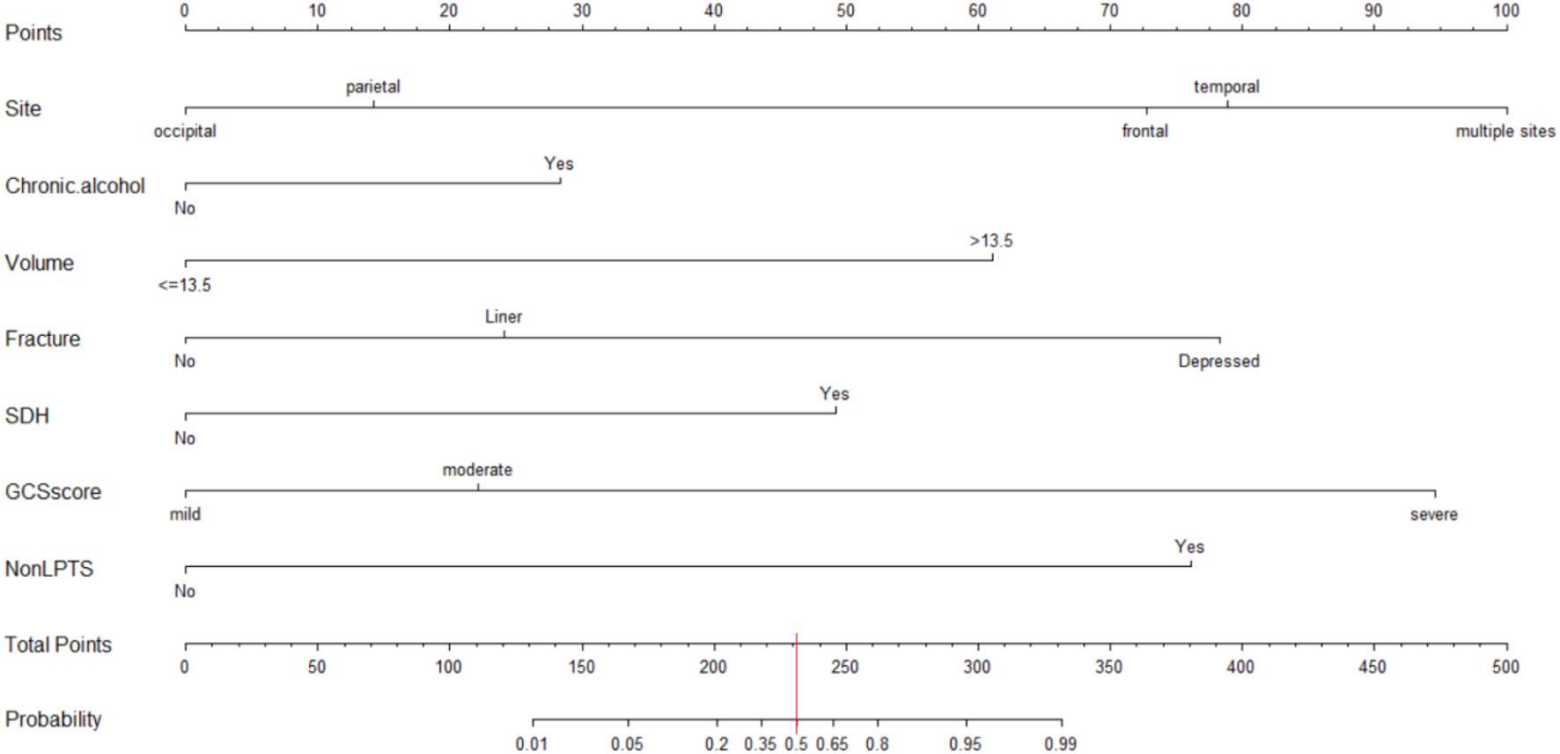
Nomogram model. *SDH* subdural hematoma, *GCS* glasgow coma scale, *LPTS* late post-traumatic seizure.

After exploring the influencing factors of PTE, it was necessary to use these factors to establish a predictive scoring system for PTE, and the statistical method chosen was Nomogram, with the following arithmetic results (Fig. 4).

The scoring system corresponding to the above line chart is shown in Table 4. For example, the patient has a contusion site on the forehead (gain 73 points) , a long drinking history (gain 28 points) , and a contusion volume > 13.5 ml (gain 61 points) , SDH(gain 49 points) , GCS score = 9分 (gain 22 points) . But, the patient did not have Fracture (gain 0 points) and Non-LPTS (gain 0 points). The PTE risk score of the patient is as follows: 73 + 28 + 61 + 0 + 49 + 22 + 0 = 233. Next, bringing the score of 233 into the chart (Fig. 4), we can see that for patients with a score of 233, the probability of developing PTE is about 53%.

**Table 4:** The corresponding scoring system table of Nomogram.

Factors		Points
Site	Occipital	0
	Parietal	14
	Frontal	73
	Temporal	79
	Multiple	100
Chronic alcohol use	No	0
	Yes	28
Volume	≤ 13.5	0
	> 13.5	61
Fracture	No	0
	Liner	24
	Depressed	78
SDH	No	0
	Yes	49
GCS score	13–15 (mild)	0
	9–12 (moderate)	22
	3–8 (severe)	95
Non-LPTS	No	0
	Yes	76

*SDH* subdural hematoma, *GCS* glasgow coma scale, *LPTS* late post-traumatic seizure.

#### Application and verification of nomogram

The corresponding relationship between the score and the probability of PTE occurrence is shown in Table 5. A cut point of 50% means that a patient has a higher than 50% probability of developing a PTE when his score is more significant than 231.

**Table 5:** The probability of prognosis corresponding to the scoring system.

Total points	Probability (%)
131	1.00
167	5.00
184	10.00
194	15.00
201	20.00
208	25.00
213	30.00
218	35.00
223	40.00
227	45.00
**231**	**50.00**
236	55.00
240	60.00
245	65.00
250	70.00
255	75.00
262	80.00
269	85.00
279	90.00
296	95.00
332	99.00

Taking 50% as the cut point, that is, the patient's score is greater than 231, which means that the patient will have PTE. The prediction accuracy of the above scoring system is 98.29%. The confidence interval of the C-index is 97.28% to 99.30%.

**Figure 5:**
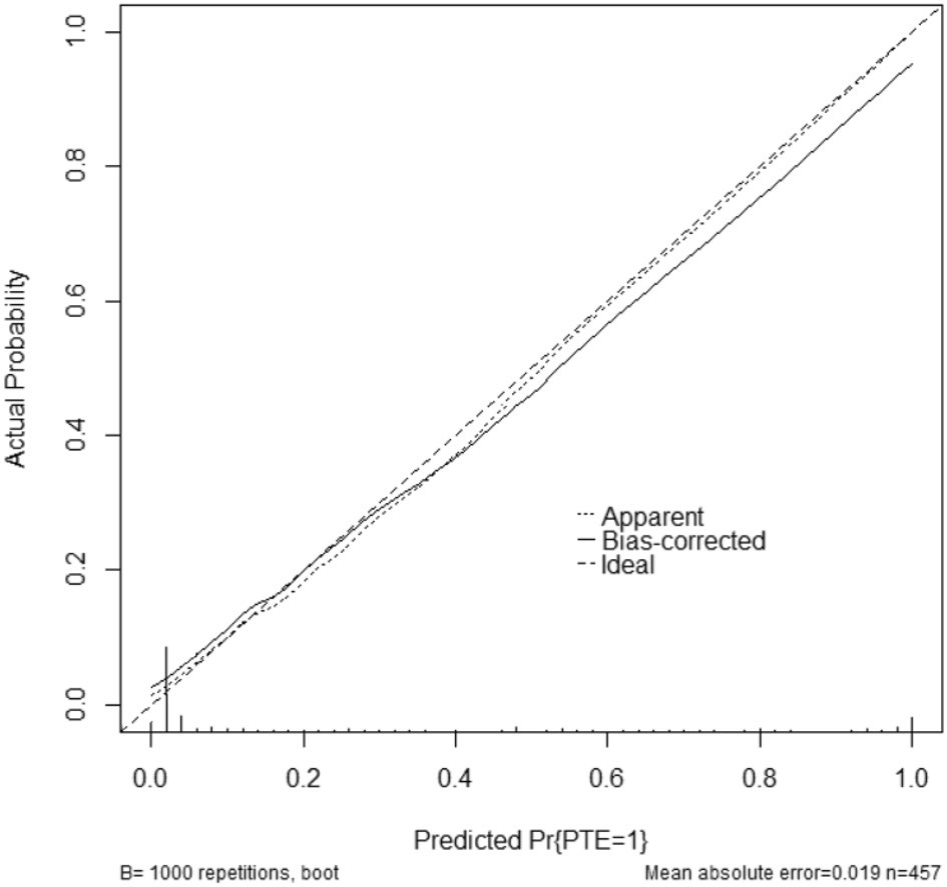
The calibration plot between the predicted results and the actual results. The prediction results (curve) are consistent with the theoretical results (diagonals), which means the accuracy of the prediction results.

**Figure 6:**
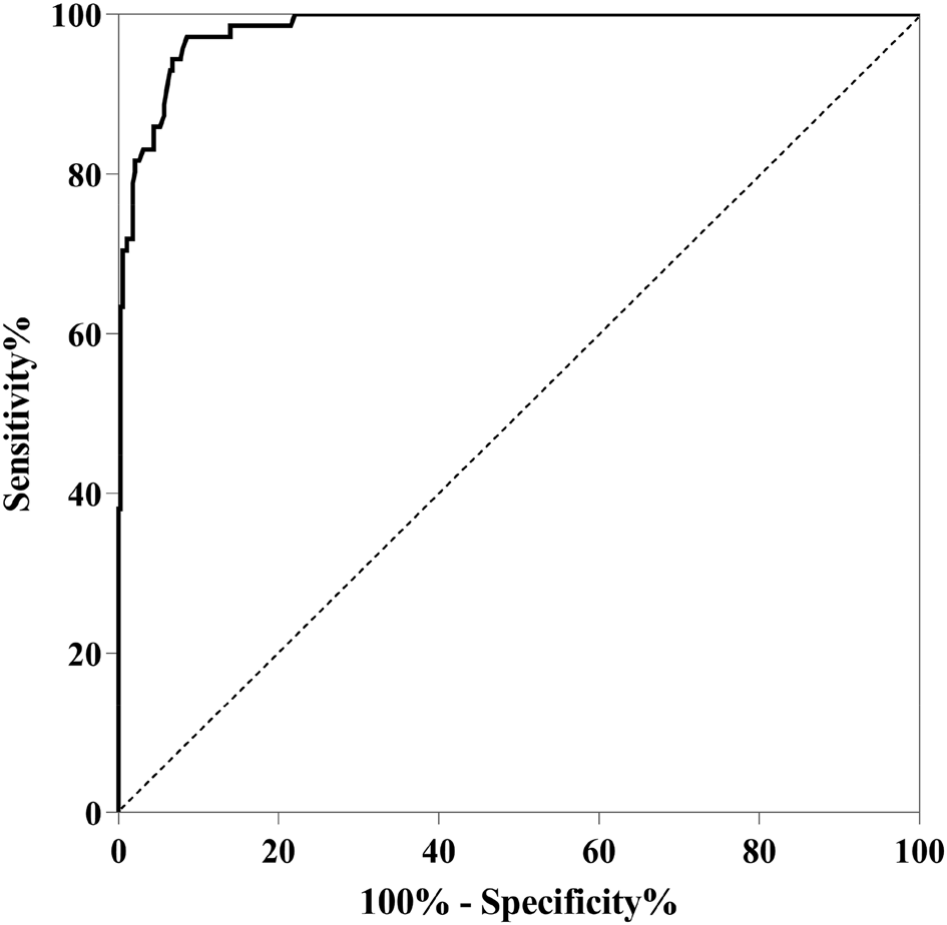
Nomogram scores were calculated for each patient in this study, and ROC curves were plotted (AUC = 0.983; 95% CI 0.966–0.993).

After constructing the Nomogram to predict the risk of PTE, we internally validated the model. Using the 1000 boot strapping method, the points obtained for each variable were summed, and the total number of points corresponding to the risk of PTE in percentage form (Fig. 4). And the calibration plot (Fig. 5) is the visualization we performed after the internal validation. In the calibration plot (Fig. 5), the X-axis represents the Nomogram prediction, and Y-axis represents the observed rate of the outcome event in the validation cohort. Furthermore, the diagonal line represents the ideal performance of the Nomogram, and it can be seen that the prediction result (curve) is basically consistent with the theoretical result (diagonal line). Its prediction accuracy C-index = 98.29%. The confidence interval of the C-index is 97.28% ~ 99.30%. In addition, we calculated the Nomogram score for each patient participating in this study. We plotted the ROC curve, yielding an AUC = 0.983 (95% CI: 0.966 ~ 0.993) (Fig. 6), further illustrating the predicted results' accuracy.

## Discussion

PTE has always been one of the most worrying complications of cerebral contusion among clinicians. Because of its disabling severe, it brings a massive burden to the patient's family and society. Some risk indicators of PTE hidden in patients' baseline information and clinical examination can indicate whether patients will be complicated with PTE, which can provide vital information for early, timely and accurate judgment of high-risk patients. By identifying high-risk groups early and paying particular attention to them, and preventing them, the impact of poor prognosis on patients' families and society can be reduced. At the same time, it is hoped that the antiepileptic treatment of PTE will be possible by studying these high-risk groups in the future. In this study, by collecting the data of patients with a cerebral contusion in Qinghai People's Hospital, we established the related factors of PTE and investigated the incidence rate. Our results revealed seven risk factors for predicting PTE occurrence: Site, Chronic alcohol use, Volume, Fracture, SDH, GCS score, and Non-LPTS. In addition, we constructed a nomogram based on these seven risk factors to predict the individual risk of PTE in clinical practice.

As we all know, the most common injury sites of brain trauma are the frontal lobe and anterior temporal lobe, which are related to the anatomical structure of the skull, but the location of the lesion is an important factor affecting the occurrence of PTE. It has been reported that PTE occurs most frequently in the temporal lobe, occasionally in the frontal lobe, and rarely in the occipital and parietal lobes^17,18^. Our results are consistent with that. The incidence of PTE in the temporal and frontal is significantly higher than that in the Parietal and occipital. The temporal lobe is not only the most frequently involved area of PTE but its medial structure is also related to human refractory PTE^18,19^. Unlike previous studies on single-site injuries, we also included multiple-site cerebral contusions (≥ 2 sites). Finally, our results suggest that multiple-site injuries are more likely to develop PTE than single-site injuries. It has been reported that the biological mechanism of PTE may involve the destruction of networks in specific areas, such as the network involving the temporal lobe, and the destruction of temporal lobe-related circuits may lead to epilepsy^17^. Therefore, we suspect that when multiple site injuries are involved, more regional network sites are destroyed, so PTE is more likely to occur. Multiple-site cerebral contusions is also one of the strongest and most statistically significant predictors of PTE in our model.

The contusion volume is essential in predicting post-traumatic seizures^3,20^. In the previous prediction model of PTE, the specific contusion volume has never been recorded. Our research is different from the previous research. For the first time, we used 3D slicer software to accurately calculate the contusion volume to predict the occurrence of PTE. We found that when the cerebral contusion volume is > 13.5 ml, the risk of PTE increases. This may be because when the volume of contusion is larger, later secondary injuries such as brain edema tend to be more severe, resulting in higher intracranial pressure and metabolic crisis, thus leading to seizures. In addition, the larger volume of contusion leads to an obvious midline shift, and the neurons between severe contusion areas are injured. After that, with the increase of contusion load, neuronal injury and apoptosis may increase, destroy neuronal circuits and make the focal area prone to discharge^21^. Some studies have also shown many processes after cerebral contusion, including necrosis, microhemorrhage, apoptosis, axonal injury, microglia proliferation, demyelination, inflammatory and oxidative stress, and later phases of neurodegeneration, regeneration, and vascular remodeling. These processes may lead to changes in the epileptic circuitry that can trigger seizures^22^. Therefore, the larger the contusion volume, the easier it is to induce epilepsy.

Since seizures are mainly related to abnormal discharges of the cerebral cortical network, post-traumatic skull fractures will cause significant damage to the cerebral cortex. At the same time, skull fracture also increases the risk of secondary brain injury after craniocerebral trauma, such as the further expansion of hematoma and further aggravation of edema. Chronic complications may also occur if the skull is an open fracture, including poor wound healing, cerebrospinal fluid (CSF) leakage, and infection. Some studies have found that depressed skull fracture will produce scars in the cerebral cortex after injury, increasing PTE risk by 50%^23^. In other studies, surgical treatment (including decompressive craniectomy and postoperative bone flap reduction) has also been associated with PTE^24^. No matter which kind of surgical treatment, it will also cause damage to the cerebral cortex, and the occurrence of related postoperative complications may further aggravate the brain damage, which increases the risk of PTE. However, this factor was insignificant in our cohort study (*P* = 0.064). One possible explanation is that patients undergoing surgery are generally patients with severe cerebral contusions who need to be referred to the intensive care unit for further active treatment. Most patients use sedatives and mechanical ventilation in the intensive care unit so that seizures may be suppressed or masked. It may also be that our data sample size is not large enough to analyze the significant relationship between different treatments and PTE. Further studies may be needed to determine the exact effects of different treatments on PTE in the future.

EDH has been reported to increase the risk of post-traumatic epilepsy^11^. SDH has also been a risk factor for post-traumatic epilepsy^16,25,26^. In our study, based on univariate analysis, the P values of EDH and SDH were 0.894 and < 0.001, respectively. Therefore, we further analyze SDH and find that SDH affects PTE, while EDH is not the risk factor affecting PTE. This may be because the injured neurons in the cerebral cortex are the origin of epileptic activity, and there is a tendency to scar the cortex in the area formed by SDH. According to the basic mechanism of cortical scar in epileptogenesis^27,28^, SDH is easy to induces seizures can be explained. In addition, compared with SDH, the brain tissue damage of EDH patients is usually less^3^, which may also be one of the reasons why EDH is not easy to induce PTE.

Previous studies have shown that the risk of epilepsy after craniocerebral trauma is closely related to the severity of head injury^3,15^. For the classification of the severity of brain trauma, some studies use LOC time to classify^29^, and some studies use GCS score to classify^11^. Considering that the GCS score already includes an assessment of the patient's state of consciousness, therefore, we used the GCS score measured at admission to classify the severity of cerebral contusion as follows: 3–8 are severe; 9–12 are moderate; 13–15 are mild^30^. Our study found that in the multivariate analysis model, moderate cerebral contusion was a predictor of PTE compared with a mild cerebral contusion, while severe cerebral contusion was a significant predictor of PTE. In general, the lower the GCS score, the more severe the brain damage, stimulating extracellular ion exchange and glutamate release, resulting in increased excitatory connections, which is more likely to induce epilepsy^31^. Moreover, the severity of cerebral contusion is closely related to the site, volume, skull fracture, and other risk factors of contusion. Therefore, the lower the GCS score, the higher the risk of seizures.

Annegers and Alan et al. ^32,33^ reported that acute epileptic seizures after trauma (defined as seizures occurring within seven days of TBI) did not increase the rate of epileptic recurrence in TBI patients. Therefore acute epileptic seizures were not a risk factor for PTE. Some studies^3,13^ believe that early-onset (within seven days) after trauma is a predictor of PTE, and secondary brain injury caused by acute onset plays a vital role in the progression of PTE and significantly increases the chance of subsequent epilepsy^3,4,13,34,35^. Due to the limited number of samples and the relatively small number of patients with acute episodes in the clinic, our study did not separate IPTS from EPTS for related factor analysis but summed up Non-LPTS (within seven days) as related factors for analysis. Finally, our results support the latter view, and we find a significant correlation between Non-LPTS and the occurrence of PTE. This may be due to early seizures and the destruction of related neuroregulatory mechanisms after injury, resulting in changes in the balance of neurons and further reorganization of neuronal circuits in systems that are already prone to epilepsy^36^. Therefore, effective prevention of IPTS/EPTS is essential to reduce PTE risk. We recommend prophylactic use of antiepileptic drug treatment for patients with a cerebral contusion in the acute phase. Although this may not reduce the incidence of PTE, it may reduce acute episodes, thereby reducing secondary brain injury and prolonging the latency of PTE (defined as the interval between the occurrence of brain injury and the onset of the first PTE), thus improving the prognosis of PTE^9^.

Studies have shown that Chronic alcohol use (defined as daily alcohol consumption for ≥ 1 year) is a significant risk factor for early PTS^26,37,38^. In our study, Chronic alcohol use was also a high-risk predictor of PTE (OR = 7.442, *P* < 0.05). In Qinghai, due to regional and cultural reasons, most local people have a habit of alcohol consumption. Most of the patients admitted to the hospital after trauma are also related to drinking. Alcohol drinking may increase the seizure threshold by acting on the γ-aminobutyric acid receptor and decrease the seizure threshold by up-regulating the N-methyl-D-aspartate receptor when drinking is stopped. Therefore, in patients with a cerebral contusion, sudden cessation of drinking (within 6–48 h) may induce seizures^39^. Through the above multivariate analysis of Non-LPTS, we speculate that chronic alcohol use increases the incidence of early PTS and, therefore, increases PTE incidence.

We discussed and analyzed the dangerous effects of Site, Chronic alcohol use, Volume, Fracture, SDH, GCS and Non-LPTS on PTE. For high-risk patients, it seems that only Chronic alcohol use can help prevent seizures by stopping drinking at a later stage, and other factors are measured after the head injury has occurred. Therefore, we can only improve the prognosis by prophylactic administration of antiepileptic drugs after head injury. For epilepsy prevention, although it is generally accepted that prophylactic antiepileptic treatment does not reduce the incidence of PTE, it may reduce acute seizures and, by reducing secondary brain injury, is expected to prolong the latency period of PTE and thus improve the prognosis of PTE. And our Nomogram may be a valuable tool for identifying these high-risk individuals.

Nevertheless, this research also has some limitations. Firstly, this study is inherently limited by retrospective as a retrospective study. Secondly, We also do not rule out the possibility that people who experience actual epileptic activity, patients and their families do not know, or misreporting is caused by memory bias. This may also cause us to underestimate the occurrence of PTE. Thirdly, The follow-up period is relatively short because the risk of PTE may exist even decades after brain contusion.

## Conclusion

This study provides an easy-to-use tool to predict PTE risk in patients with a cerebral contusion. Early identification of high-risk groups helps optimize the classification and management of patients with a cerebral contusion. In this study, based on the baseline and clinical data of patients, Site, Chronic alcohol use, Volume, Fracture, SDH, GCS, and Non-LPTS were screened and analyzed as the significant factors affecting PTE occurrence. In addition, we included the specific contusion volume into the model prediction for the first time to improve the PTE prediction model for patients with a cerebral contusion. However, as this study is a retrospective study, and the sample size is limited, a multicenter, prospective large sample design study is still needed to supplement and verify the prediction model.

## Data Availability

The datasets generated and/or analyzed during the current study are not publicly available due to the Qinghai Provincial People's Hospital regulations that the datasets are confidential and will not be shared, but are available from the corresponding author on reasonable request.

## Abbreviations

TBI: Traumatic brain injury
PTE: Post-traumatic epilepsy
PTS: Post-traumatic seizure
IPTS: Immediate post-traumatic seizure
EPTS: Early post-traumatic seizure
LPTS: Late post-traumatic seizure
Non-LPTS: IPTS + EPTS
EDH: Epidural hematoma
SDH: Subdural hematoma
LOC: Loss of consciousness
GCS: Glasgow coma scale
ICP: Intracranial pressure
C-G/L: Cerebrospinal fluid glucose/cerebrospinal fluid lactate ratio
ICI: Intracranial infections
GOS: Glasgow outcome scale
SAH: Subarachnoid hemorrhage
TVS: Third ventricle status
EEG: Electroencephalography
